# Influence of Self-Efficacy on Cancer-Related Fatigue and Health-Related Quality of Life in Young Survivors of Childhood Cancer

**DOI:** 10.3390/ijerph19031467

**Published:** 2022-01-27

**Authors:** Masayo Saito, Izumi Hiramoto, Michihiro Yano, Arata Watanabe, Hideya Kodama

**Affiliations:** 1 Department of Nursing, School of Health Science, Akita University Graduate School of Medicine and Faculty of Medicine, Akita 010-8543, Japan; msaito@hs.akita-u.ac.jp (M.S.); hirahira2918@yahoo.co.jp (I.H.); 2 Department of Pediatrics, Akita University Hospital, Akita 010-8543, Japan; yanomi@doc.med.akita-u.ac.jp; 3 Department of Pediatrics, Nakadori General Hospital, Akita 010-8577, Japan; 8914arata@gmail.com

**Keywords:** self-efficacy, cancer-related fatigue, health-related quality of life, childhood cancer survivors, structural equation model

## Abstract

This study aims to elucidate how self-efficacy influences cancer-related fatigue and health-related quality of life (HRQoL) in young survivors of childhood cancer. Forty-six young survivors (age range, 8–18 years) of childhood cancer who were currently in complete remission completed measures for self-efficacy (Pediatric General Self-Efficacy Scale (PedsSE)), cancer-related fatigue (Cancer-related Fatigue Score (CRFS)), and HRQoL (Pediatric Quality of Life Inventory 4.0 Generic Core Scale, Pediatric Quality of Life Inventory (PedsQL)). Structural relationships between the PedsSE and CRFS or PedsQL, including the effects of potential demographic or clinical confounders, were examined by machine learning random forest algorithms and structural equation modeling. According to the distribution of the PedsQL, six survivors with PedsQL < 70 were determined to have compromised HRQoL (referred to as “low-PedsQL survivors”). The random forest model identified six variables for the prediction of the CRFS, with the PedsSE being the most important, and eight variables for the distinction of low-PedsQL survivors, with the CRFS being the most and the PedsSE the third most important variable. The structural equation model indicated that a direct influence of the PedsSE on the PedsQL was less detectable (β = −0.049), whereas an indirect influence of the PedsSE on the PedsQL via the CRFS was evident (β = 0.333). The model explained 51% of the variation of the CRFS and 28% of the variation of the PedsQL. The PedsSE was strongly correlated with “altered mood” in the subclass of the CRFS (r = −0.470), and “altered mood” was strongly correlated with the PedsQL (r = 0.737). In conclusion, self-efficacy is a major determinant of cancer-related fatigue and influences HRQoL via cancer-related fatigue in survivors of childhood cancer. The main pathway from self-efficacy to HRQoL is thought to be via the emotional aspect of cancer-related fatigue. However, unlike adult survivors of cancer, self-efficacy for young survivors may not contribute much to self-management behaviors that maintain HRQoL.

## 1. Introduction

As the survival rates for childhood cancers have improved remarkably over the past few decades, the number of long-term survivors continues to increase [1,2,3]. Inevitably, the long-term psychophysiological consequences of surviving childhood cancer patients have attracted the attention of many health-care professionals [4]. Since childhood cancer occurs during critical stages of growth and development, the impacts of disease and treatment on quality of life (QoL) might be more complicated and sustained in children than in adults. Therefore, young survivors may encounter more challenging situations in many areas of their school lives, which could lead to behavioral problems and social adjustment disorders [5,6,7].

Health-related QoL (HRQoL) is an individual’s perceived physical and mental health over time as assessed by multiple domains related to physical, mental, emotional, and social functioning and is commonly used as a measure in various fields of health-care research [8]. Because cancer survivors are required to self-manage the consequences of cancer and its treatment, self-management competence plays a vital role in the maintenance of health and well-being. Such self-management competence is enhanced through the perception of self-efficacy, an individual’s belief in their capacity to execute appropriate action to face environmental challenges [9]. It is well known that among adult survivors of cancer, greater self-efficacy is conductive to engaging in healthier behaviors, which lead to better HRQoL [10,11,12,13]. However, whether this remains true for young survivors of childhood cancer remains unclear because their health-care management is often left to others, such as their parents. To our knowledge, only one study has reported the influence of self-efficacy on HRQoL in childhood cancer survivors [14]. They reported that general self-efficacy was included in significant influential factors of HRQoL by the multiple regression analysis although the impact of general self-efficacy was relatively smaller than those of the other significant sociodemographic factors, such as current age, currently not attending school, having many uncomfortable symptoms, and daily life difficulties.

Cancer-related fatigue is characterized by excessive and persistent exhaustive symptoms that interfere with daily activity and function in patients with cancer and often persist for months or years after treatment [15,16]. For survivors of childhood cancer, fatigue is often a central issue that exacerbates HRQoL and requires adequate self-management behaviors [17,18]. Fatigue is a very subjective complaint, and greater self-efficacy has often been reported to have a strong negative association with perception of fatigue in adult patients with cancer [19,20]. Psychological resilience is the ability to revert from psychological distortions and is understood to be a positive process that allows people to adapt well in the face of adversity [21]. For most cancer survivors, the experience of cancer and its treatment is remembered as a process of recovering from severe hardship, and psychological resilience becomes part of their QoL [22]. Self-efficacy is a major determinant of psychological resilience in cancer patients [23,24], and thus, positive effects of self-efficacy on cancer-related fatigue or QoL could be achieved by enhancing psychological resilience [25].

Some theoretical models have been proposed and tested to clarify the relationships among self-efficacy, cancer-related fatigue, and HRQoL in adult patients with cancer under various situations [26,27,28,29,30]. The findings of these previous studies have suggested that self-efficacy directly and indirectly influences HRQoL and that cancer-related fatigue mediates the influence of self-efficacy on HRQoL. Various models have provided insight that interventions to improve self-efficacy could contribute to the alleviation of fatigue and improved QoL in this population. However, such a model has not been tested for survivors of childhood cancer, and the effectiveness of interventions that target self-efficacy in survivors of cancer remains unconvincing [31].

A better understanding of the impact of self-efficacy on cancer-related fatigue and HRQoL could provide insights into interventions for health-care professionals who are required to intervene in the treatment of young survivors of cancer whose HRQoL is compromised because of poor self-management behaviors. Therefore, this study aims to elucidate how self-efficacy influences cancer-related fatigue and HRQoL in young survivors of childhood cancer.

## 2. Materials and Methods

### 2.1. Participants

The study participants were young survivors of childhood cancer who had been treated for cancers at each of two general hospitals they attended regularly for routine health checkups. First, we asked pediatricians at the hospitals to recommend patients who could participate in the study. The inclusion criteria were as follows: current age between 8 and 18 years, previously diagnosed as having childhood cancer and received chemotherapy, no cancer treatment for at least the previous 1 year, and currently in complete remission. Only those who had sufficient ability to answer a questionnaire survey were included. We explained the research purpose and outline of this study to the recommended patients and their parents. The content of the explanation included that participation in the study was voluntary, that there would be no disadvantage for deciding not to participate in the study, and consent to withdraw was guaranteed at all times. Final agreement to participate in the study was confirmed by signing the consent form.

A total of 46 young survivors of childhood cancer were enrolled in the study. Clinical treatment data were obtained from the survivors’ medical charts. Table 1 shows the demographic and clinical characteristics of the survivors of childhood cancer. The diagnosis of blood cancer for 33 survivors included leukemia for 26 (56.5%) and malignant lymphoma for seven (15.2%). The diagnosis of solid cancers for 13 survivors included brain tumors for four (8.7%) and non-brain tumors for nine (19.6%). Treatment was performed by chemotherapy alone for 29 patients (63.0%) and a combination of chemotherapy, surgery, and radiation for four (21.7%).

### 2.2. Questionnaire Survey

Two self-efficacy measures were used in this study. The self-efficacy of young survivors of childhood cancer (age 8–12 years) was assessed using the General Self-Efficacy Scale for Children—Revised, which was developed for Japanese schoolchildren in 2009 by Fukui et al. [32]. This scale is composed of 18 items divided by two dimensions: sense of security (9 items) and spirit of challenge (9 items). Each item is scored from 1 (“not at all true”) to 4 (“completely true”), and the total score ranges from 18 to 72, with higher scores indicating greater self-efficacy. Cronbach’s α among Japanese primary schoolchildren was 0.77 [32]. The self-efficacy of young survivors of childhood cancer aged 13 years or older was assessed using the widely used self-efficacy measure, the General Self-Efficacy Scale [33]. This scale is composed of 10 items, with each scored from 1 (“not at all true”) to 4 (“completely true”). The total score ranges from 10 to 40, with higher scores indicating greater self-efficacy. In samples from 23 nations, Cronbach’s α ranged from 0.76 to 0.90, with the majority in the high 0.80 s [34].

The cancer-related fatigue of survivors of childhood cancer was assessed via a questionnaire (referred to as the Cancer-related Fatigue Score (CRFS)) used in a previous study by Nagai et al. [30]. The CRFS was developed to measure fatigue in survivors of leukemia aged >8 years without any cancer treatment for at least the previous 1 year. The CRFS is composed of 12 items that were designed to assess three dimensions of symptoms: physical fatigue (feel tired, want to lie down, forceless, still tired after night’s sleep), decreased function (lack of energy, make easy mistakes, sleepy in the daytime, unrefreshing wake-up), and altered mood (lack of concentration, irritated, anxious about the body, depressed), each of which includes four items. Each item is scored from 0 to 3 (from 0, “not at all” to 3, “almost every day”), and the total score ranges from 0 to 36. Higher scores indicate stronger symptoms of fatigue. Cronbach’s α was reported to be between 0.75 and 0.88 for the total and each of the three fatigue dimension scores in both a patient and a control group [35].

The HRQoL of survivors of childhood cancer was assessed using the Pediatric Quality of Life Inventory (PedsQL) 4.0 Generic Core Scale [36]. The PedsQL is composed of 23 items that belong to one of the four following domains: physical functioning (8 items), emotional functioning (5 items), social functioning (5 items), and school functioning (5 items). For each item, the participants were asked about the frequency of a problem during the past 1 month. Responses were given on a 5-point Likert scale, ranging from 0, “never” to 4, “almost always.” Then, the scores were reverse-scored and linearly transformed to a 0–100 scale (0 = 100, 1 = 75, 2 = 50, 3 = 25, 4 = 0), with higher scores representing a better QoL. The reliability of the Japanese version of the PedsQL has been validated (Cronbach’s α > 0.70), except for the “school functioning” subscale in schoolchildren and adolescents aged 8–18 years [37].

### 2.3. Statistics

JMP (ver. 14; SAS Institute Inc., Cary, NC, USA) was used for the basic statistical analysis. Means ± standard deviations were used to express continuous variables, and the Shapiro–Wilk test was used to examine the normality of distributions. Because the majority of variables was not normally deviated with a low sample size, nonparametric statistical methods were used to examine group differences or correlations. In all statistical analyses, cases with a *p*-value < 0.05 were considered significant. Random forest models for regression or binomial classification were constructed to predict the CRFS or distinguish participants from survivors associated with the low PedsQL using Minitab (R)19 statistical software (Kozo Keikaku Engineering Inc., Tokyo, Japan). For each model, the important variables for prediction or distinction were selected and ranked according to mean decrease Gini. The effectiveness of the regression model was assessed by R-squared and the root-mean-square error (RMSE), and the average loglikelihood and the area under the curve of the receiver operating characteristic (ROC) curve were used for a binomial classification model. A structural equation model was employed to examine the direct and indirect influences of self-efficacy on HRQoL using IBM SPSS Amos 28 (IBM Japan, Tokyo). The proposed model was assessed by widely accepted fit measures, including the chi-square statistic divided by the degrees of freedom (CMIN/DF < 3 acceptable, <2 excellent), a goodness-of-fit index (GFI > 0.90 acceptable, >0.95 excellent), a comparative fit index (CFI > 0.90 acceptable, >0.95 excellent), and the root-mean-square error of approximation (RMSEA < 0.08 acceptable, <0.05 excellent).

## 3. Results

Table 2 shows the distributions of the measures on self-efficacy, CRFS, and HRQoL (PedsQL) in survivors of childhood cancer. Both measures on self-efficacy for survivors aged between 8 and 12 years (General Self-Efficacy Scale for Children—Revised) and for survivors aged 13 years or more (General Self-Efficacy Scale) were distributed widely without left- or right-side deviation. To integrate these two measures, we calculated a deviation value for each patient for each measure separately and presented this as the Pediatric General Self-Efficacy Scale (PedsSE). The CRFS was distributed without left- or right-side deviation, while the PedsQL was distributed with right-side deviation.

**Figure 1:**
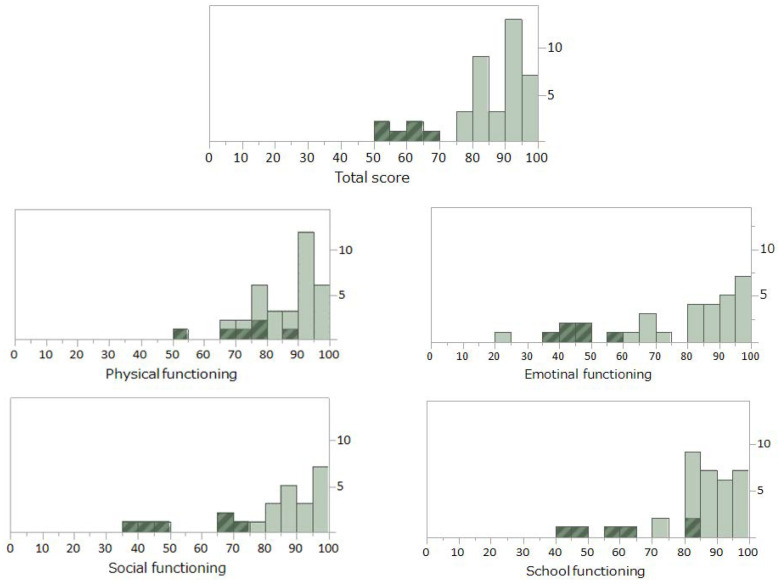
Histograms presenting the distribution of a measure for health-related quality of life (a total score and scores for subscales of PedsQL) in childhood cancer survivors (*n* = 46). Six survivors defined as “low-PedsQL survivors” were shown by dark columns with diagonal lines.

Figure 1 shows histograms presenting the distribution of total and subscale PedsQL scores for survivors of childhood cancer. The distribution of the total score (see upper panel) deviated to the right side, and the values of 40 survivors were clustered above 75 points. Because the mean of the total score of the PedsQL in healthy Japanese children of these ages has been reported to be 82.7 ± 11.6 [37], the scores in these 40 survivors were considered to be within the normal range. The total scores of the other six survivors were distributed below 70 points and clearly separated from the other survivors in the normal range (shown by dark columns with diagonal lines). Several surveys on HRQoL in survivors of childhood cancer have reported that most survivors are psychologically healthy although certain groups of survivors show compromised HRQoL [38,39,40,41]. Our PedsQL distribution supported these observations; therefore, we determined these six survivors as having compromised HRQoL (hereafter, referred to as “low-PedsQL survivors”). As for histograms presenting the distributions of subscale PedsQL scores (see middle and lower panels), low-PedsQL survivors were located in the low-score range, with the exception of their scores on “physical functioning”. No significant differences in demographic or clinical variables (listed in Table 1) were found between the two groups of survivors. However, the PedsSE or CRFS in the low-PedsQL survivors was significantly lower or higher than that of the other survivors (42.7 ± 6.5 vs. 51.1 ± 9.9, *p* = 0.025, or 17.3 ± 4.3 vs. 10.0 ± 6.3, *p* = 0.010).

**Figure 2:**
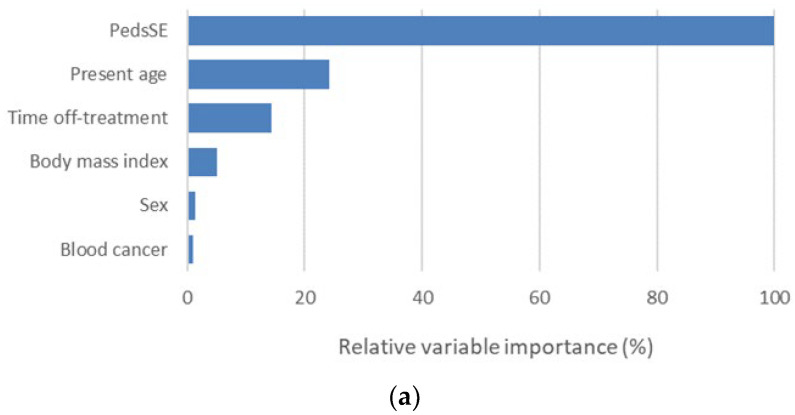

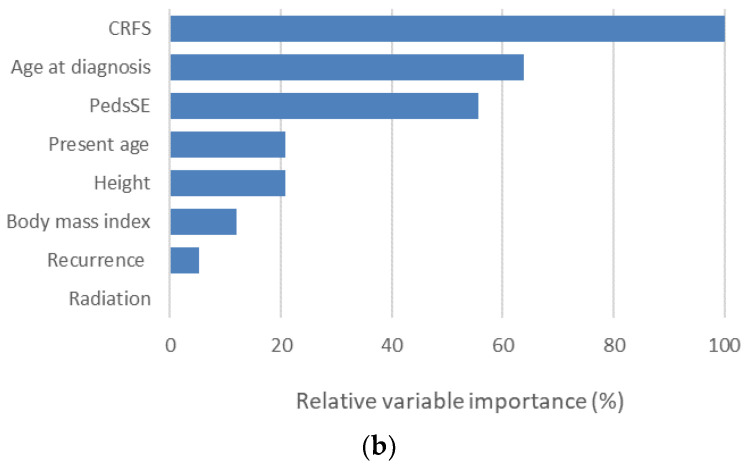
Ranking of important variables identified by random forest algorithm of regression (**a**) for prediction of cancer-related fatigue (CRHS) or binomial classification and (**b**) for distinction of low-PedsQL survivors in childhood cancer survivors (*n* = 46).

For the purpose of identifying the variables associated with the CRFS or PedsQL, we constructed random forest models instead of a conventional multivariate linear regression model because this machine learning model has better detection capability when a relatively large number of potential confounders, many of which may have multicollinearity, are tested together. Prior to the analysis, demographic and clinical variables were tested to see if they could predict the PedsSE; however, no effective model was constructed (R-squared = 0.0%, RMSE = 10.3). Figure 2 shows important variables selected by the random forest regression model (a) for prediction of the CRFS or binomial classification model (b) to distinguish low-PedsQL survivors. The random forest model for prediction of the CRFS, which was constructed using available demographic and clinical variables and the PedsSE, identified a total of six variables as important for prediction, with the PedsSE being the most important (R-squared = 17.8%, RMSE = 5.85). The random forest model for the distinction of low-PedsQL survivors, which was constructed using available demographic and clinical variables, the PedsSE and CRFS, identified a total of eight variables as important variables, with the CRFS being the most important and the PedsSE being the third most important (average loglikelihood = 2.06, area under the ROC curve (95% confidence interval) = 0.60 (0.305–0.896)).

**Figure 3:**
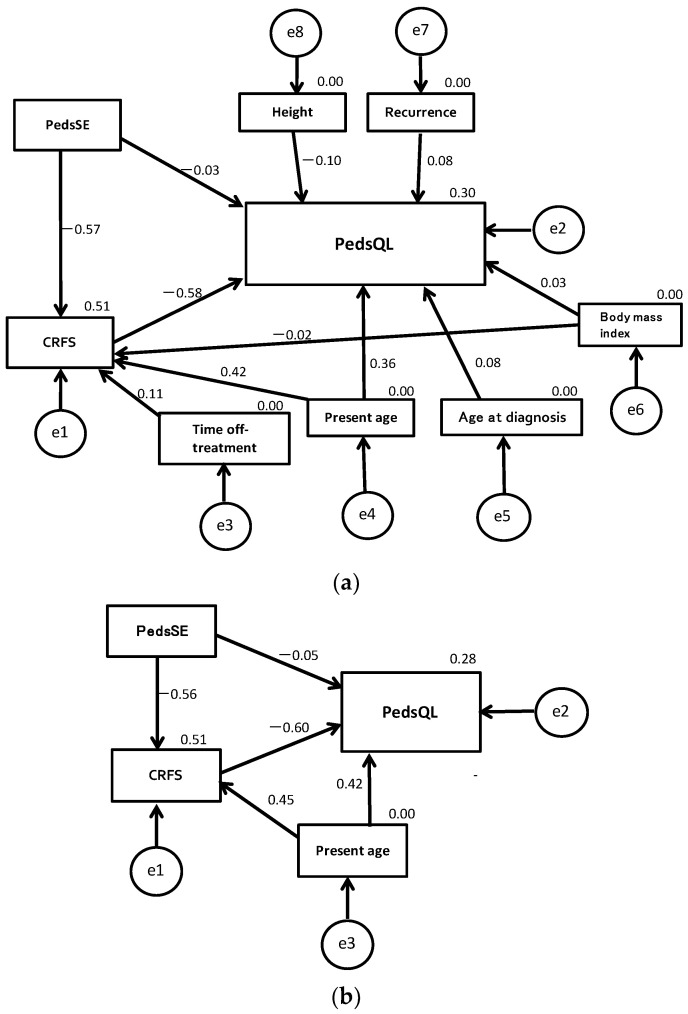
Structural equation models examining the influence of self-efficacy on cancer-related fatigue and health-related quality of life in childhood cancer survivors (*n* = 46). The model (**a**) were constructed including all possible demographic and clinical confounders. The model (**b**) were constructed excluding all nonsignificant possible confounders (including “present age“ only).

Figure 3 shows a structural equation model examining the effect of self-efficacy on cancer-related fatigue and HRQoL in survivors of childhood cancer. Figure 3a shows a model that assumes direct and indirect influences of self-efficacy on HRQoL (low-PedsQL survivors = 0, other survivors = 1), including the influence of possible demographic and clinical confounders selected by random forest models (radiation, sex, and blood cancer were not included because of their very low importance). However, the goodness-of-fit for this model was extremely low (CMIN/DF = 8.92, GFI = 0.785, CFI = 0.159, RMSEA = 0.419). Therefore, the demographic and clinical variables except for “present age” were removed to modify the model because their paths to self-efficacy or HRQoL were nonsignificant. Figure 3b shows a modified model for which all fit measures were excellent (CMIN/DF = 0.073, GFI = 0.999, CFI = 1.000, RMSEA < 0.001). In this model, the standardized path coefficients (β) from the PedsSE to PedsQL, PedsSE to CRFS, and CRFS to PedsQL were −0.049 (*p* = 0.765), −0.556 (*p* < 0.001), and −0.598 (*p* < 0.001), respectively. Although the direct influence of the PedsSE on the PedsQL was almost indetectable, the indirect influence of the PedsSE on the PedsQL through the CRFS was evident (β = 0.333). “Present age” had a significant positive impact on both the CRFS (β = 0.450, *p* < 0.001) and PedsQL (β = 0.416, *p* = 0.006). According to the squares of multiple correlation coefficients, this model explained 51% and 28% of the variation of the CRFS and PedsQL, respectively.

Table 3 shows Spearman’s rank correlation coefficients between each subscale of the CRFS and PedsSE or the PedsQL subscales in survivors of childhood cancer. The PedsSE had relatively strong negative correlations with “decreased function” (r = −0.523) and “altered mood” (r = −0.470), and “altered mood” had a relatively strong negative correlation with the PedsQL (r = −0.737). Therefore, the main pathway from self-efficacy to HRQoL was thought to be mediated by the emotional aspect of cancer-related fatigue.

## 4. Discussion

A theoretical model to examine the relationships among self-efficacy, cancer-related fatigue, and HRQoL in cancer patients was first proposed by Hoffman et al. [26]. They reported that self-efficacy had a positive effect on physical function status and served as a mediator between fatigue and physical function status in patients with cancer who were undergoing chemotherapy. Since then, similar models have been tested for adult patients with cancer in different situations. Haas [27] tested an introductory model of cancer-related fatigue, self-efficacy, physical activity, and QoL in patients with breast cancer who were currently receiving treatment and concluded that the model explained 31% of the variance in self-efficacy. Phillips and McAuley [29] longitudinally tested a model examining self-efficacy and health status as potential mediators of the relationship between physical activity and QoL in survivors of breast cancer and concluded that physical activity indirectly influences QoL across time via self-efficacy and health status. Buffart et al. [28] examined the mediating mechanisms of an exercise intervention on QoL in cancer survivors (≥3 months posttreatment, 57% breast cancer) and found that the beneficial effect of the intervention was mediated by physical activity, self-efficacy and mastery, and subsequent reductions in fatigue and distress. Chen et al. [30]. reported that self-efficacy had a direct and indirect effect on QoL in patients with resected lung cancer and that cancer-related fatigue could mediate the relationship between self-efficacy and QoL. The relationships of these measures are considered to be basically bidirectional, and the models were constructed depending on the purpose of studies that the researchers hypothesized. To our knowledge, our study is the first to examine such a model in survivors of childhood cancer, and self-efficacy was set as a starting point of the path diagram to elucidate the influence of self-efficacy on the other two factors [30].

Greater self-efficacy is associated with more successful health-maintenance behaviors in children with chronic disease, including type 1 diabetes [42], obesity [43], asthma [44], and functional constipation [45]. However, for young survivors of childhood cancer, the influence of self-efficacy on HRQoL appears to be limited. The PedsSE was the most important determinant of the CRFS and explained 51% of the variance. However, the PedsSE had a less direct influence on the PedsQL. Since the CRFS was the most important determinant of the PedsQL, the influence of self-efficacy on HRQoL was considered to be mediated by cancer-related fatigue. However, the impact of the CRFS on the PedsQL was only 28% even if it included the influence of confounding age factors. The reason for this limited influence may be explained by the fact that many survivors of childhood cancer basically leave the management of their lives to their parents and that their self-efficacy is still immature and in a developmental stage. Self-efficacy was a major determinant of the perception of fatigue in survivors of childhood cancer and was implicated as an important internal factor associated with phycological resilience against the perception of fatigue. However, because of the relatively low impact of the CRFS on the PedsQL, cancer-related fatigue may not be a central issue regarding HRQoL for many school-age survivors. In connection with this issue, the effect of “present age” as a confounding factor on these relationships was interesting. In this study, “present age” had positive impact on both the CRFS and PedsQL although the CRFS had a negative impact on the PedsQL. This seemingly contradictory relationship can be explained by the fact that relatively young survivors are more likely to realize compromised QoL when they feel fatigued as compared with older survivors.

Fatigue is a multidimensional symptom that includes physical, psychological, and emotional aspects, and the CRFS consists of three subscales corresponding to these three dimensions of fatigue [35]. The PedsSE had a relatively strong negative correlation with “altered mood” on the CRFS subscale, and “altered mood” had a relatively strong negative correlation with the PedsQL. Therefore, the main pathway from self-efficacy to HRQoL could be mediated by the emotional aspect of cancer-related fatigue, which suggests that when a patient with childhood cancer feels fatigued, the existence of emotional factors, such as depression and anxiety, should be suspected in the background. In adult patients with cancer, exercise as a nonpharmacological intervention has been effective to alleviate cancer-related fatigue [46,47], and the effectiveness of the intervention is associated with perceived self-efficacy [48,49]. Cancer-related fatigue in children improves over time, and increased physical activity is associated with less cancer-related fatigue [50]. However, the effectiveness of physical exercise training interventions in patients with childhood cancer remains unconvincing, and this type of intervention might be not as effective as that in adult patients with cancer [31]. If mental health status plays a key role in fatigue among survivors of childhood cancer, psychological interventions that increase self-efficacy might be more effective [51].

This study had several limitations. First, the effectiveness of our self-efficacy measure was not validated in the pediatric population. Because the widely used self-efficacy measure did not assume young subjects under the age of 12 years, we used another measure for young survivors under 12 years old. Therefore, in this study, the self-efficacy of the patients was evaluated using different measures; however, we thought that it was possible to integrate the two measures using a deviation value for each patient because the distributions of both measures were similar. However, since the predictive validity of this integrated measure has not been verified, it is considered to be a limitation of this study. Second, the significant impacts of some confounding factors could not be detected because of the small sample size. For example, the elapsed time after the end of treatment might have had a significant impact on cancer-related fatigue. Furthermore, the most important factor in increasing self-efficacy is past achievements and successful experiences, and survivors of childhood cancer can more positively recognize past experiences if they survive longer. Therefore, the elapsed time after the end of treatment may also have a positive impact on self-efficacy. However, such influence could not be well verified because of the large variability in the present age and the age of diagnosis among our survivors despite the relatively small sample size. Third, there might be unmeasured confounding factors regarding the relationships among our measures. For example, the QoL of survivors may be largely affected by familial factors, such as financial status, working status of parents, and presence of siblings, but this study did not collect these data. Furthermore, such family factors and the strength of parent–child bonding may be involved in the process by which childhood cancer survivors develop self-efficacy as they grow up. However, such a process has not been fully explained yet, and this issue is suggested as a future topic of research in this field.

In conclusion, this study clarified how self-efficacy influences cancer-related fatigue and HRQoL in young survivors of childhood cancer. Self-efficacy is a major determinant of cancer-related fatigue and influences HRQoL via cancer-related fatigue. The main pathway from self-efficacy to HRQoL is possibly mediated by the emotional aspect of cancer-related fatigue. However, the influence of self-efficacy on HRQoL appears to be limited because the direct positive influence of self-efficacy on HRQoL was less detectable, and cancer-related fatigue had a relatively small impact on HRQoL. Unlike adult survivors of cancer, self-efficacy for young survivors may not contribute much to self-management behaviors that maintain HRQoL.

Finally, as the “take-home message” of this study, we would like to emphasize the importance of providing support to young survivors, focusing on the emotional aspects of cancer-related fatigue. It is necessary to continuously screen for anxiety and depression symptoms, evaluate any difficulties in their school lives, and collect information to lead to timely intervention. In addition, although a strengthening of parent–child bonding occurs through the experience of cancer, it may inhibit the development of self-efficacy in the survivors. If such a situation is predicted, efforts should be made to encourage the self-care competence of survivors while watching the closeness of their family relationships. Approximately 10–15% of young survivors of childhood cancer suffered from poor QoL, and healthcare professionals should consider aggressive interventions to help such survivors.

## Figures and Tables

**Table 1:** Demographic and clinical characteristics in childhood cancer survivors (*n* = 46).

	Distribution	Range
Demographic characteristics		
Age (years)	13.3 ± 3.1	8–18
Sex (male)	26 (56.5)	-
Height (±standard deviation)	−0.09 ± 1.20	−3.50–3.10
Body mass index (kg/m^2^)	18.2 ±3.0	14.6–27.3
Clinical characteristics		
Age at diagnosis (years)	4.1 ± 4.4	0–14
Duration of hospital stay (months)	86.8 ± 12.8	1–52
Time off-treatment (months)	101 ± 50	12–206
Diagnosis		
Blood cancer	33 (71.7)	-
Solid cancers	13 (28.3)	-
Treatment		
Chemotherapy	46 (100)	-
Surgery	10 (21.7)	-
Radiation	9 (20.0)	-
Stem cell transplantation	3 (6.5)	-
Recurrence of cancer	5 (10.9)	-

Distributions are expressed as means ± standard deviations or frequencies (percentages).

**Table 2:** Distributions of measures on self-efficacy, cancer-related fatigue, and health-related quality of life in childhood cancer survivors (*n* = 46).

	Distribution	Range
General Self-Efficacy Scale		
General Self-Efficacy Scale for Children—Revised	53.1 ± 9.4	38–70
(For survivors of 8–12 years old, *n* = 18)		
General Self-Efficacy Scale	28.2 ± 5.1	20–37
(For survivors of 13–18 years old, *n* = 28)		
Pediatric General Self-Efficacy Scale (PedsSE)	50.0 ± 9.9	33.9–67.9
(For all survivors, *n* = 46)		
Cancer-related Fatigue Score (CRFS)		
Total score	10.9 ± 6.5	0–25
Physical fatigue	3.8 ± 2.8	0–9
Decreased function	4.7 ± 2.5	1–9
Altered mood	2.5 ± 2.4	0–10
Pediatric Quality of Life Inventory (PedsQL)		
Total score	86.8 ± 12.8	54.3–100
Physical functioning	89.3 ± 11.2	53.1–100
Emotional functioning	82.3 ± 21.4	20–100
Social functioning	89.1 ± 16.4	35–100
School functioning	86.1 ± 14.2	40–100

Distributions are expressed as means ± standard deviations.

**Table 3:** Correlations between each subscale of the CRFS and PedsSE or the PedsQL subscales in childhood cancer survivors (*n* = 46).

	CRFS		
	Physical Fatigue	Decreased Function	Altered Mood
PedsSE	−0.384	**−0.523**	−0.47
PedsQL			
Total score	**−0.587**	−0.484	**−0.737**
Physical functioning	**−0.569**	−0.391	**−0.665**
Emotional functioning	−0.436	−0.411	**−0.659**
Social functioning	−0.383	−0.309	−0.381
School functioning	−0.441	−0.437	**−0.675**

Values are Spearman’s rank correlation coefficients. Bold values are high correlation values (absolute values > 0.5).

## Data Availability

Data sharing is not applicable for this article.
